# Lipedema in a male patient: familial evidence and clinical response to nutritional intervention – case report

**DOI:** 10.1590/1677-5449.202502102

**Published:** 2026-10-09

**Authors:** Michelle Kaefer, Bruna Candia Piccoli, Sabrina Fontana de Andrade, Laura Eduarda de Oliveira, Ighor Seiji Okumura Tioda, Luisa Buhse Pasqualoto, Clóvis Paniz, José Antonio Mainardi de Carvalho

**Affiliations:** 1 Universidade Federal de Santa Maria – UFSM, Santa Maria, RS, Brasil.; 2 Universidade Estadual de Campinas – Unicamp, Campinas, SP, Brasil.

**Keywords:** lipedema, male patient, gluteofemoral adiposity, prolactin, case report, lipedema, paciente masculino, adiposidade gluteofemoral, prolactina, relato de caso

## Abstract

Lipedema is a chronic adipose tissue disorder that predominantly affects women, although some rare cases do occur in men. We report the case of a 32-year-old man with symmetrical, lower-limb fat accumulation, foot sparing, pain, easy bruising, and a positive family history of lipedema. His first-degree female cousin exhibited the classic phenotype. Dual-energy X-ray absorptiometry demonstrated greater fat accumulation in the gynoid than in the android and visceral regions, reinforcing a predominantly gluteofemoral adiposity pattern. The patient underwent a 7-month personalized nutritional intervention consisting of a moderately hypocaloric, high-protein, low-glycemic diet with vitamin D supplementation and regular physical activity. This intervention was associated with reductions in anthropometric measurements and decreased pain scores on a Visual Analogue Scale. Laboratory tests revealed a preserved hypothalamic–pituitary–gonadal axis, except for mild idiopathic hyperprolactinemia that normalized during follow-up. This case highlights lipedema in men and the potential benefits of early lifestyle-based interventions.

## Resumo

O lipedema é um distúrbio crônico do tecido adiposo que afeta predominantemente mulheres, embora ocorram casos raros em homens. Relatamos o caso de um homem de 32 anos com acúmulo simétrico de gordura nos membros inferiores, dor, preservação dos pés, tendência a equimoses e histórico familiar positivo para lipedema. Sua prima de primeiro grau apresentava fenótipo clássico. A absorciometria por dupla emissão de raios X demonstrou maior acúmulo de gordura na região ginoide do que nas regiões androide e visceral, reforçando um padrão de adiposidade predominantemente gluteofemoral. O paciente foi submetido a intervenção nutricional personalizada por sete meses, baseada em dieta hipocalórica moderada, hiperproteica e de baixo índice glicêmico, associada à suplementação de vitamina D e à prática regular de atividade física. Essa intervenção resultou em redução das medidas antropométricas e diminuição dos escores de dor na Escala Visual Analógica. Os exames laboratoriais revelaram preservação do eixo hipotálamo-hipófise-gônadas, exceto por uma hiperprolactinemia idiopática leve, que se normalizou durante o acompanhamento. Este caso destaca a ocorrência de lipedema em homens e os potenciais benefícios de intervenções precoces baseadas em mudanças no estilo de vida.

## INTRODUCTION

Lipedema is a chronic disease predominantly affecting women that has features of both adipose disorder and lymphatic dysfunction, causing reduced lymphatic transport capacity and lasting symptoms.^1,2^ Lipedema in men is rare and few cases have been published, although clinical recognition and descriptive detail have increased recently.^3-8^ To contextualize this still uncommon presentation, Table 1 summarizes published male lipedema cases with available clinical data, comparing disease stage, comorbidities, diagnostic approaches, treatments, and the main contributions of each report. Although reports of cases in men remain scarce, features similar to those seen in women have been observed, including positive family history, possible hormonal and genetic influences, and clinical response to dietary strategies such as ketogenic and low-carbohydrate diets.^9-11^

**Table 1:** Comparative table of published cases of male lipedema.

**Study / Year**	**N**	**Age (y)**	**Clinical Stage**	**Comorbidities**	**Diagnostic assessment**	**Treatment**	**Highlights**
Chen et al., 2004^3^	1	20	Not classified (functional)	None	Clinical, MRI, normal lymphoscintigraphy	Tumescent liposuction	First English-language case; emphasized MRI for diagnosis
Bertlich et al., 2021^4^	1	62	Stage II, with lipolymphedema	Type 1 DM, hypertension, alcohol use	Clinical, ultrasound, histology, lab tests	Tumescent liposuction (8 L removed)	Reported successful liposuction in an advanced male case
Cadart et al., 2021^5^	2	55 and 62	Stage I–II and lipedema + lymphedema	Klinefelter syndrome, obesity, diabetes (in one case), venous insufficiency	Clinical, ultrasound, hormone panel	Compression (ineffective), supportive care	Linked lipedema to genetic hypogonadism (karyotype 47, XXY)
Pereira de Godoy et al., 2022^6^	1	53	Lipedema + systemic lymphedema	Oncologic surgery, RT/CT	Multi-segment bioimpedance (3-year follow-up)	Godoy Method® (lymphatic therapy)	First to document progression to systemic lymphedema by bioimpedance
Alim & Suryana, 2022^7^	1	39	Stage III + systemic lymphedema	Morbid obesity (BMI ~54), mild hypertension	Clinical, perimeter, bioimpedance	NSAIDs, antihypertensives, spontaneous weight loss	Emphasizes diagnostic consideration in obese males with leg edema
Amato et al., 2025^8^	5	31–58 (mean: 42.6)	Stage II–III	80% endocrine disorders, 60% family history, 1 HLA-DQ2/DQ8+	Clinical, multifrequency bioimpedance or DXA (in 2 cases)	GLP-1, lisdexamfetamine, ketogenic diet, exercise.	Largest case series to date; proposed HLA link and gluten-free diet response
This study (2026)	1	32	Stage I–II	History of adolescent obesity, mild monomeric hyperprolactinemia, and positive family history	Clinical, DXA, bioimpedance, hormone panel	Personalized nutritional intervention, exercise.	Familial evidence, clinical and laboratory evidence of improvement was observed after a 7-month nutritional intervention

Methodological Note: This comparative table summarizes all published case reports and series describing male patients diagnosed with lipedema, identified through a targeted literature search of PubMed, Scopus, and Google Scholar (2004–2025). Only peer-reviewed articles providing clinical and diagnostic details were included. The comparison covers clinical stage, comorbidities, diagnostic tools, therapeutic interventions, and unique clinical or scientific insights. Differences in classification criteria, diagnostic tools, and treatment follow-up limit direct comparability, but the compilation highlights the evolving understanding of male lipedema and its potential hormonal, genetic, and lymphatic underpinnings. Abbreviations: BMI = Body Mass Index; CT = Chemotherapy; DM = Diabetes Mellitus; DXA = Dual-Energy X-ray Absorptiometry; GLP-1 = Glucagon-Like Peptide-1; HLA = Human Leukocyte Antigen; MRI = Magnetic Resonance Imaging; NSAIDs = Nonsteroidal Anti-Inflammatory Drugs; RT = Radiotherapy.

This study was conducted in accordance with the Declaration of Helsinki. It was approved by the local Ethics Committee (protocol number 5.986.428) and both participants provided written informed consent before inclusion.

## CASE REPORT

A 32-year-old male patient was identified during an epidemiological study of adipose tissue disorders conducted by the Universidade Federal de Santa Maria (UFSM) Laboratory of Applied Clinical Analysis Research (LAPACA), in Santa Maria, RS, Brazil. He presented with progressive, disproportionate fat accumulation in the lower body, particularly in the hips, thighs, and infra-abdominal region. The patient reported that this body pattern had been present since childhood, with a noticeable increase beginning at age 8. By age 17, he weighed 98 kg and measured 1.76 m in height (BMI: 31.6 kg/m^2^), showing pronounced enlargement of the lower limbs, buttocks, and lower abdomen, contrasting with a relatively lean upper body.

By age 24, he had reduced his weight to 66 kg (BMI: 21.3 kg/m^2^) and reached 10% body fat through strict diet and exercise. However, gluteofemoral fat persisted, indicating localized resistance to weight loss. Since adolescence, he had experienced chronic burning pain and tenderness in the lower limbs. Pain was initially rated 5 on the Visual Analogue Scale (VAS; 0 = no pain, 10 = worst imaginable pain). Other symptoms included mild, non-pitting edema, easy bruising, muscle cramps, and increased touch sensitivity. He also reported reduced libido.

Physical examination revealed symmetric adipose deposition in the thighs and hips, with palpable subcutaneous nodules and irregular skin texture. Feet and ankles were spared, with no signs of venous insufficiency or lymphedema. The Stemmer sign was tested by attempting to pinch a skin fold over the dorsal surface of the second toe. This maneuver was negative, as the examiner was able to lift the skin fold, which is inconsistent with clinically evident lymphedema. Figure 1 shows clinical images of the patient.

**Figure 1:**
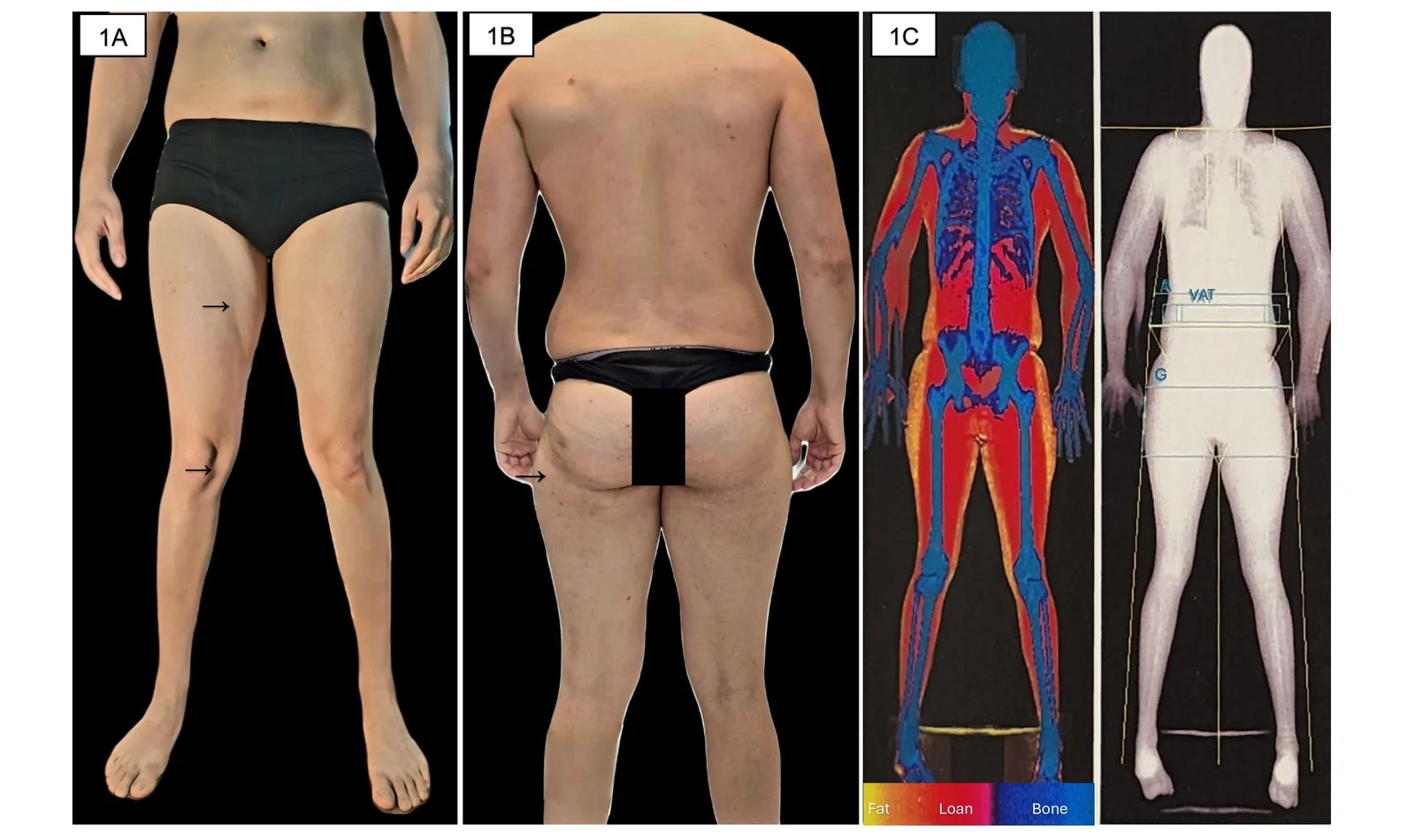
Case presentation and Dual-energy X-ray absorptiometry images. **A)** Fat accumulation on the inner thighs and knees. **B)** Predominant fat distribution in the gynoid region (hips and upper thighs). **C)** Dual-energy X-ray absorptiometry (DXA) scan showing fat compartments. VAT: Visceral adipose tissue; A: Android fat; G: Gynoid fat.

The patient reported a similar fat distribution pattern among maternal relatives, including his mother and a female cousin. A first-degree cousin, the daughter of his mother’s brother, shared the same phenotype, consented to participate, and underwent clinical evaluation. She exhibited classical lipedema features consistent with established diagnostic criteria (Figure 2). Overall, the clinical findings and family history substantiated the diagnosis of early-stage (I–II) type 2 lipedema, characterized by symmetrical fat accumulation from the hips to the knees with pain and tenderness. Written informed consent for the publication of clinical images was obtained from both the patient and his relative.

**Figure 2:**
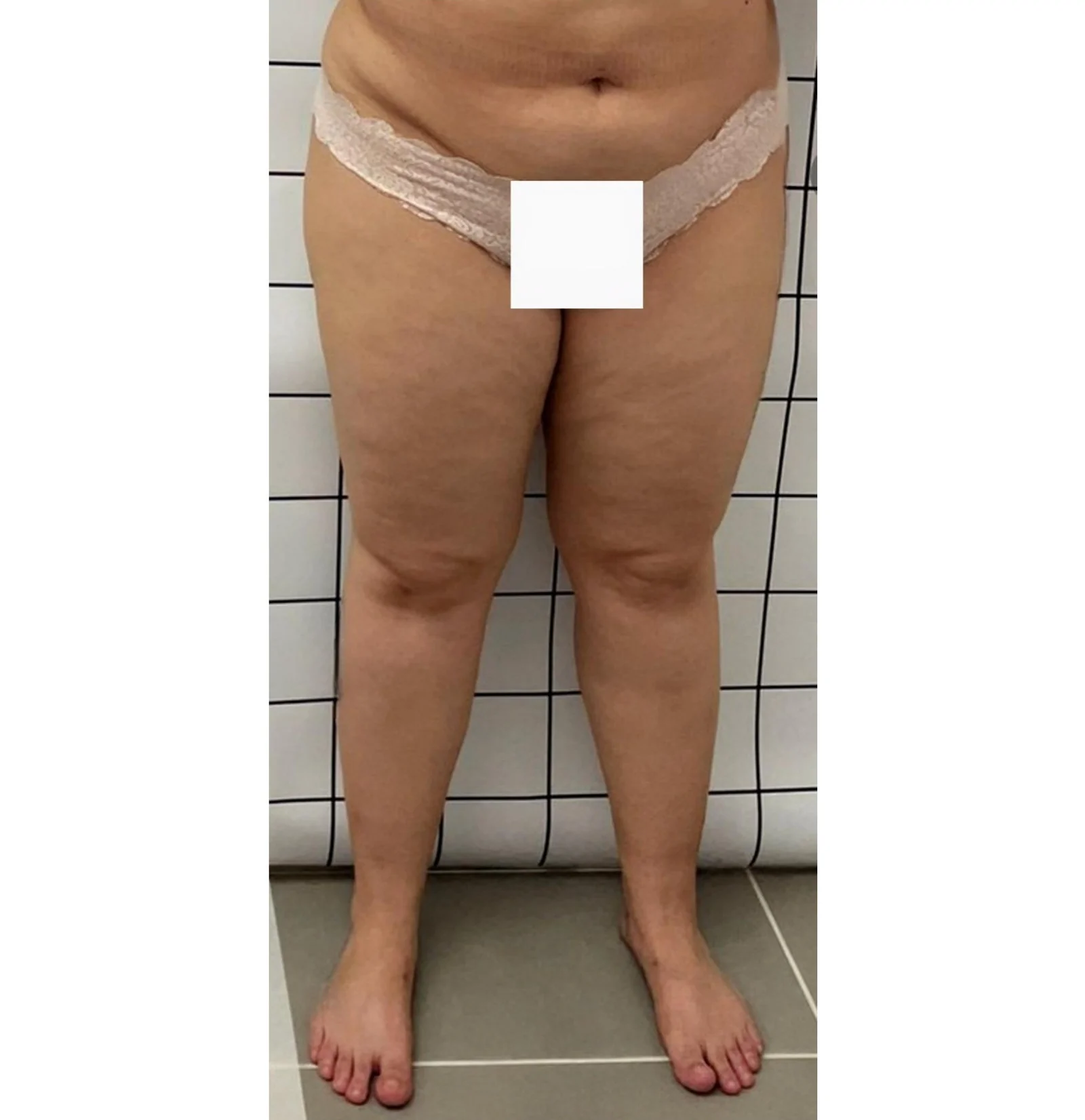
First-degree female cousin of the male patient with a lipedema diagnosis. The patient’s cousin, who shares the same maternal lineage, presents a classic lipedema phenotype with symmetric enlargement of the lower limbs, sparing the feet, and a negative Stemmer sign. Based on clinical evaluation, the case is consistent with type II, stage 2 lipedema, involving buttocks, hips, and thighs with moderate skin surface irregularity. Body Mass Index: 28.4 kg/m^2^. Waist-to-Height ratio: 0.44.

Initial laboratory evaluation revealed elevated prolactin (22.05 ng/mL; reference 4.04–15.2), prompting endocrinological assessment. The patient was not using medications known to increase prolactin. To rule out analytical interference, the test was repeated after 30 days and yielded consistent results in both undiluted serum (22.72 ng/mL) and at 1:100 dilution (22.20 ng/mL), ruling out the hook effect.^12^ Polyethylene glycol (PEG) precipitation showed 87% recovery, confirming true monomeric hyperprolactinemia.

Systemic causes of fat redistribution and edema were excluded, including hypothyroidism, Cushing’s syndrome, kidney and liver diseases, chronic venous insufficiency, HIV, rheumatologic disorders, and heart failure. Blood pressure was 128/78 mmHg, and heart rate was 51 bpm after 10 minutes at rest. Laboratory tests showed normal albumin, preserved renal and hepatic function, and no relevant rheumatologic alterations (Table 2). Semen analysis revealed normal volume, motility, morphology, and sperm vitality.

**Table 2:** Baseline laboratory findings.

**Parameter**	**Results**	**Normal Range**
Creatinine (mg/dL)	1.07	0.55-1.30
Serum sodium (mmol/L)	143.8	136-145
Serum potassium (mmol/L)	4.4	3.5-5.1
Serum chlorine (mmol/L)	104	98-107
Uric acid (mg/dL)	5.7	3.5-8.5
AST (U/L)	24	17-59
ALT (U/L)	33	Less than 50
Total protein (g/dL)	8.2	6.4-8.2
Albumin (g/dL)	4.8	3.4-5.0
Albuminuria (mg/dL)	0.17	0.10-3.0
HbA1c (%)	4.9	Less than 5.7%
Gamma-GT	15	15-73
Serum iron (μg/dL)	136	33-193
C-Reactive Protein (mg/dL)	0.14	0.00-0.30
Ferritin (ng/mL)	70.40	17.9-464
Rheumatoid factor (U/mL)	5.39	0.00-18.0
Creatine phosphokinase (U/L)	204	26-308
Lactate dehydrogenase (U/L)	145	100-190
Serum phosphorus (mg/dL)	3.3	2.5-4.9
Serum magnesium (mg/dL)	2.0	1.8-2.4
Serum calcium (mg/dL)	9.2	8.5-10.1
IgG (mg/dL)	1529	681-1648
IgM (mg/dL)	182	48-312
IgA (mg/dL)	242	87-474
C3 (mg/dL)	120	90-207
C4 (mg/dL)	23	17.4-52.2
Parathyroid hormone (pg/mL)	27.53	12-65
Thyroglobulin (ng/mL)	7.97	3.5-77
Cortisol (μg/dL)	18.87	4.82-19.5
Insulin (μIU/mL)	5.68	2.3-26
Dehydroepiandrosterone (ng/mL)	4	1.3-9.3
Serum zinc (μg/dL)	65.8	70-120
Serum selenium (μg/dL)	104	20-190

HbA1c = Glycated hemoglobin; AST = Aspartate aminotransferase; ALT = Alanine aminotransferase; IgG = Immunoglobulin G; IgM = Immunoglobulin M; IgA = Immunoglobulin A; C3 = Complement component 3; C4 = Complement component 4.

Endocrine and autoimmune tests showed normal thyroid function, negative anti-thyroglobulin and anti-thyroid peroxidase antibodies, and an overall preserved hormone profile, including luteinizing hormone (LH), testosterone, and growth hormone (GH) within reference ranges (Tables 2 and 3). Follicle-stimulating hormone (FSH) levels were slightly below the lower reference limit in both evaluations, but this isolated finding was not accompanied by other clinical or laboratory evidence of hypothalamic–pituitary–gonadal axis dysfunction. There were no clinical signs of pituitary dysfunction or neurological symptoms, such as persistent headache, visual field defects, galactorrhea, or signs of hypogonadism.

**Table 3:** Baseline and follow-up assessments of laboratory parameters (6 months) and multi-segment bioimpedance analysis (7 months)

**Parameter**	**First assessment**	**Second assessment**	**Normal range**
**Laboratory analysis**			
Fasting glucose (mg/dL)	90	78	74–99
Total cholesterol (mg/dL)	178	158	< 190
HDL cholesterol (mg/dL)	60	56	>40
LDL cholesterol (mg/dL)	104.7	87	< 130
White blood cells (/mm^3^)	6640	5490	3600–11000
Hemoglobin (g/dL)	15.4	15.9	12.8–17.8
Prolactin (ng/mL)	22.72	10.8	4.04–15.2
Estradiol (pg/mL)	22.97	20.4	6.1–91.9
TSH (μIU/mL)	2.80	2.32	0.27–4.2
Free T3 (pg/mL)	3.16	3.15	2.3–4.2
Free T4 (ng/dL)	1.32	1.37	0.93–1.71
GH (ng/mL)	0.10	<0.05	0.030–2.47
FSH (μIU/mL)	3.21	3.91	4.6–12.4
LH (μIU/mL)	6.75	4.64	4.0–8.6
Progesterone (ng/mL)	0.28	0.47	0.2–1.4
Total testosterone (ng/dL)	534.7	687	330–805
Free testosterone (ng/dL)	13.65	12.67	3.03–14.80
Ferritin (ng/mL)	70.40	50.70	17.9–464
Vitamin B12 (pg/mL)	506	662	211–911
Vitamin D (ng/mL)	22.9	33.2	>30
**Bioimpedance analysis**			
Weight (kg)	82.5	77.9	–
Height (cm)	176	176	
Body mass index (kg/m^2^)	26.6	25.1	–
Waist circumference (cm)	87	88	
Hip circumference (cm)	108	99.5	
Waist-to-height ratio	0.49	0.50	
Subcutaneous fat (%)	17.1	14.5	–
Leg fat mass (kg)	6.0	5.0	–
Leg lean mass (kg)	20.6	20.4	–
Trunk fat mass (kg)	10.2	7.9	–
Trunk lean mass (kg)	27.3	27	–
Arm fat mass (kg)	2.4	1.7	–
Arm lean mass (kg)	7.1	7.1	–

TSH = Thyroid-stimulating hormone; GH = Growth hormone; FSH = Follicle-stimulating hormone; LH = Luteinizing hormone.

Follow-up evaluation included repeat laboratory tests after 6 months of nutritional intervention and repeat bioimpedance, body circumferences, and anthropometric assessment after 7 months (Table 3). During this period, the patient adhered to a nutritional plan with supplementation of 2000 IU of vitamin D daily. The nutritional intervention was based on a moderate calorie deficit (300–500 kcal/day below the estimated energy requirements), combined with a high-protein diet providing 1.5 g of protein per kg of body weight, and a low-glycemic-index dietary pattern. The dietary plan prioritized consumption of minimally processed foods, including whole grains, fruits, vegetables, lean protein sources, and healthy fats, while reducing the intake of refined carbohydrates and ultra-processed foods. Clinical follow-up was conducted at monthly consultations throughout the intervention period, during which adjustments were made to the dietary plan and calorie intake as needed. Adherence to the dietary plan was monitored during follow-up visits based on the patient’s self-report and clinical evaluation conducted by the clinical team. He also engaged in regular physical activity, including resistance training, walking, and martial arts three to four times per week.

After 7 months, he showed marked clinical and metabolic improvement. Weight had decreased by 4.6 kg, BMI reduced from 26.6 to 25.1 kg/m^2^, and hip circumference had shrunk from 108 to 99.5 cm, indicating gluteofemoral fat loss. His subcutaneous fat percentage fell from 17.1% to 14.5%, with reductions involving legs, trunk, and arms, while his lean mass remained stable. There were reductions in fasting glucose (90→78 mg/dL), total cholesterol (178→158 mg/dL), and LDL (104.7→87 mg/dL) and prolactin normalized (22.72→10.8 ng/mL). Levels of vitamins D and B12 increased and the white blood cell count decreased slightly. Perceived pain decreased from 5 to 3–4 on the VAS, although this score may be influenced by physical activity, which can cause musculoskeletal discomfort unrelated to the adipose disorder.

Overall, findings suggest metabolic status and fat distribution both improved after nutritional intervention combined with regular physical activity. DXA was performed approximately 45 days after the initial clinical evaluation, rather than at the true baseline; this timing should be considered when interpreting body composition findings (Table 4).

**Table 4:** Body composition obtained by dual-energy X-ray absorptiometry.

**Variable**	**Value**
Total body mass (kg)	79.591
Total body fat mass (kg)	20.619
Arm fat mass (kg)	2.462
Trunk fat mass (kg)	8.528
Leg fat mass (kg)	8.480
Total body lean mass (kg)	58.972
Arm lean mass (kg)	8.178
Trunk lean mass (kg)	28.624
Leg lean mass (kg)	17.586
Gynoid fat mass (kg)	4.341
Android fat mass (kg)	1.278
Estimated visceral adipose tissue (g)	237
Leg fat mass (kg)/total body fat mass (kg) ratio	0.4204

## DISCUSSION

This case report presents clinical, biochemical, and imaging findings supporting a diagnosis of lipedema in a male patient, a rare presentation of a condition that predominantly affects women. The patient presented symmetrical lower limb fat accumulation associated with pain and tenderness, foot sparing, and a negative Stemmer sign, features commonly associated with lipedema and less typical of lymphedema. According to the Brazilian Consensus Statement on Lipedema published by the Brazilian Society of Angiology and Vascular Surgery (SBACV),^13^ lipedema diagnosis is primarily clinical and should be based on patient history, physical examination, and exclusion of differential diagnoses. Although lymphoscintigraphy could have provided additional confirmatory evidence, functional imaging of the lymphatic system was not available in this case. Therefore, while the clinical findings and positive family history were highly suggestive of a diagnosis of lipedema, the possibility of lymphatic involvement or overlap with lymphedema cannot be completely ruled out.

As described in large female cohorts,^10^ the familial pattern indicates potential autosomal dominant inheritance with variable expressivity and incomplete penetrance. The presence of lipedema in a male patient and his first-degree female cousin (his maternal uncle’s daughter) corroborates this pattern. Recent genomic studies have identified rare variants in hundreds of genes, without a single causal locus, suggesting an oligogenic model with marked heterogeneity.^14^ Over 80% of women with lipedema report a positive family history, with paternal inheritance in 32%.^10^

Comprehensive evaluation ruled out systemic causes of leg edema and enlargement. There were no signs of heart failure, oncologic disease, or protein-losing conditions. Laboratory tests showed normal albumin (4.8 g/dL), normal renal and hepatic function, and no anemia or inflammation. Rheumatologic markers including rheumatoid factor, immunoglobulins, and complement were unremarkable and HIV testing was negative. The patient denied gastrointestinal symptoms or malabsorption suggestive of intestinal protein loss. These results excluded differential diagnoses such as nephrotic syndrome, chronic liver disease, venous insufficiency, HIV-associated lipodystrophy, and inflammatory disorders.

Prolactin-secreting pituitary adenomas are the main cause of hyperprolactinemia in men, typically resulting in hypogonadism and reduced libido.^15^ Prolactin measurements can help guide etiological assessment, although mild elevations are non-specific.^16^ Values up to 100 ng/mL are commonly associated with non-tumoral causes or microprolactinomas, whereas levels above 250 ng/mL suggest prolactinoma, and those exceeding 500 ng/mL are highly indicative of macroprolactinoma.^12,16^ After excluding secondary causes, pituitary magnetic resonance imaging (MRI) is recommended, although small lesions may not always be detectable.^17^

In the present case, prolactin was only mildly elevated (~1.5× the upper limit of normal) in the context of a preserved hypothalamic–pituitary–gonadal axis and absence of clinical features suggestive of pituitary disease. Therefore, immediate imaging was deferred in favor of clinical and laboratory follow-up, during which prolactin normalized in parallel with clinical improvement.

Prolactin is also produced locally in adipose tissue. Lean individuals release more subcutaneous prolactin than obese patients, but most remains bound to proteoglycans such as heparin, limiting systemic diffusion. Thus, the contribution of adipose-derived prolactin to serum levels remains uncertain.^18,19^

Activated monocytes and macrophages within inflamed adipose depots can also secrete extrapituitary prolactin.^20^ Lipedema is characterized by macrophage infiltration, stromal inflammation, and extracellular matrix remodeling.^21,22^ In this context, prolactin may be involved in modulating the local inflammatory microenvironment. The observed reduction in gluteofemoral fat and body weight may have attenuated local inflammation, potentially contributing to the normalization of prolactin levels seen during follow-up. However, a causal relationship cannot be established based on the present findings.

Although not diagnostic, dual-energy X-ray absorptiometry (DXA) helps quantify fat distribution and support clinical suspicion. The scan showed a leg fat-to-total fat ratio of 0.420, exceeding the 0.383 threshold proposed in a female cohort.^23^

However, its applicability to men remains uncertain due to sex-related differences in fat distribution. Additionally, the patient presented a predominance of gynoid fat (4.34 kg) over android fat (1.28 kg), along with low visceral fat (237 g). No follow-up DXA was performed, but serial bioimpedance showed reductions in leg fat (−1.0 kg), trunk fat (−2.3 kg), and subcutaneous fat percentage (17.1 → 14.5%). Hip circumference decreased from 108 to 99.5 cm, while the waist remained stable (87 → 88 cm), yielding a waist-to-height ratio of 0.50.^24^

These changes followed a hypocaloric, high-protein, low-glycemic, anti-inflammatory diet with vitamin D, previously associated with improvement in female lipedema.^11^ The intervention was accompanied by weight loss and symptomatic relief, including reduced pain, as well as improvement in bruising frequency and lower-limb heaviness. Regular physical activity was maintained throughout follow-up and likely contributed to the overall clinical improvement alongside the nutritional strategy.

Follow-up tests showed decreases in fasting glucose, total and LDL cholesterol, and white blood cell count, with increases in vitamin D, vitamin B12, hemoglobin, and testosterone, while thyroid and gonadal hormones remained stable. Although follow-up documented consistent improvements in anthropometric measures, body composition, laboratory parameters, and symptoms, a longer follow-up period would be valuable to determine whether these improvements are sustained over time. Despite its rarity, this case of lipedema in a male patient underscores the need for increased clinical awareness of this underdiagnosed condition, emphasizing the dynamic between genetics, adipose tissue biology, and inflammation.

This case shows that lipedema should not be ruled out based on sex, particularly in men with early-onset, symmetrical lower-body adiposity and persistent gluteofemoral fat resistant to routine weight loss strategies. Most published male cases describe advanced-stage lipedema with lipo-lymphedema or generalized type III patterns. In contrast, our report documents a young male patient with early-stage gluteofemoral lipedema, emphasizing the importance of identifying atypical early presentations. Objective tools such as DXA and segmental bioimpedance were valuable for diagnosis and follow-up, while the favorable response to nutritional therapy suggests that early intervention may improve symptoms and quality of life.

## Data Availability

The data supporting this study are available upon request from the corresponding author, JAMC.
